# Supervisor experiences of extended clinical placements in optometry: a mixed methods study

**DOI:** 10.1186/s12909-022-03918-2

**Published:** 2022-12-09

**Authors:** Jacqueline M Kirkman, Sharon A Bentley, Ryan J Wood-Bradley, Craig A Woods, James A Armitage

**Affiliations:** 1 Deakin Optometry, School of Medicine, Deakin University, Waurn Ponds, Australia; 2 School of Optometry and Vision Science, Queensland University of Technology, Kelvin Grove, Australia; 3 School of Optometry and Vision Sciences, University of New South Wales, Sydney, Australia

**Keywords:** Clinical placements, Students, Supervisors, Rural, Education

## Abstract

**Background:**

In Australia, optometry students have traditionally undertaken their clinical training in short-block rotations at University-led teaching clinics in metropolitan locations. Demand for clinical placements is growing as the number of optometry students steadily increases. As such, universities and clinical education providers must look for more diverse methods of student placement. Extended clinical placements in community-based settings are one alternative: a model similar to the longitudinal clerkships in medicine. This study aimed to explore the experience of extended clinical placements from the perspective of the optometrists who supervised students. It also sought to determine whether there were differences in views between metropolitan and rural practitioners.

**Methods:**

This mixed methods study included a survey and interviews with optometrists who had previously supervised Deakin University optometry students on an extended 26-week (2 x 13-weeks) clinical placement. Lines of enquiry focused on; the benefits and challenges associated with extended placements; areas for improvement; duration of the placement; and willingness to supervise further students. Interviews were transcribed verbatim and analysed using Braun and Clarke’s 6 step method of thematic analysis with a qualitative descriptive approach.

**Results:**

Supervisors felt that hosting a student prompted greater reflective practice and critical appraisal of clinical decisions. The extended nature of the placement was thought to foster greater immersion in the clinical setting and community for the students and establish a stronger relationship between supervisor and student. Supervisors recognised the importance of role-modelling and mentoring the next generation of optometrists however noted that taking on a student was a sizeable commitment. Willingness to host a student was not dependent on the supervisor’s location (rural vs metropolitan) *p* = 0.57. However, interviews uncovered motivations that were unique to supervisors residing in rural locations, such as succession planning.

**Conclusion:**

Overall, supervisors were positive about the value of student extended clinical placement in optometry and felt that it was a fulfilling and professionally beneficial experience. Lack of time and financial remuneration were the key downsides highlighted. Schools of optometry might carefully consider engaging in discussion about the duration of such placements, but 26 weeks was considered appropriate by supervisors.

**Supplementary Information:**

The online version contains supplementary material available at 10.1186/s12909-022-03918-2.

## Introduction

Training of students in the health professions involves undertaking clinical placements. During these placements students develop clinical skill proficiency and learn how to confidently and professionally interact with patients (1, 2). However, placements come with the unique challenge of providing students with a valuable educational experience, while promoting optimal patient care, without adversely impacting the clinical setting in which the placement is being held (3, 4). In Australia, clinical training of nursing, allied health and medical students has traditionally occurred in metropolitan locations in university-led teaching facilities or public hospitals. As such providers of placements seek diversity in placement experiences (1-3, 5). For instance, the opportunity to experience practice in rural localities (1). Furthermore, as the number of university students across health disciplines are increasing, universities and clinical education providers look for alternative methods of student placement (5).

In the context of optometry in Australia, historically the majority of student clinical placements have been conducted in university or non-government-organisation clinics (3, 6). Yet, across Australia there are only a small number of publicly and government funded clinics in existence. Instead, optometrists in Australia predominantly practice in privately-owned, community-based practices. Given it makes reasonable sense to train students in an environment that they are most likely to work in, some Australian optometry programs are increasingly relying on community-based practices to host students on clinical placement.

Historically, optometry student placements have run in short block rotations, varying in duration from a few days to a few weeks (3). This is changing, as some optometry programs are incorporating a model which includes an extended clinical placement in a community-based practice; a model similar to that of the longitudinal integrated clerkships (LIC) seen in the medical field. Extended placements have been suggested as a way to overcome the downsides of shorter placements (7). Namely they allow for greater continuity in patient care, supervision and mentorship, and location (1). These placements need to be viable and effective for both students and supervisors (8). However, it is unknown what impact placing students in a community practice may have on the business in which they are placed and the supervising optometrist overseeing care delivery.

Anecdotally, in Australia the impression has been that optometrists are averse to the concept of hosting students for placement in community practice, especially for longer duration placements. In 2016, Bentley et al., surveyed Australian optometrists on their willingness to host a student for an extended period of placement (3). While they found moderate support for the concept of extended placements, the optometrists surveyed were concerned there would be a lack of time, financial support and space to host a student (3). A 2017 investigation which described the experience of supervising medical students in a LIC as compared to the traditional rotation curriculum model reported that supervisors found they developed closer, more meaningful relationships with the students which aided teaching advanced skills and the delivery of more constructive feedback (9). Overall, the experience was found to be a more positive one for the supervisors and made them more likely to return to the program to supervise in future years (9).

Data from studies of medical placements suggest that a greater continuity occurs in longer placements as they enable students to integrate into practice life and subsequently students are able to reciprocate benefit to the practice (1, 2, 8). Extended clinical placements are thought to foster the ideal conditions in which social and spatial connectedness to a rural community can develop (10-12). This is particularly pertinent for the optometry profession where there is currently an uneven distribution of practitioners, with a bias towards practice in metropolitan areas (13). This is not a problem unique to Australia as uneven distributions of optometrists are also evident in other countries (14). Rural practice owners in Australia have historically had difficulty recruiting optometrists. Many regions instead rely on a fly-in fly-out workforce of optometrists. For this reason, it has been thought that rural practitioners may be more willing to host students for longer duration placements, as students may be more likely to return to practice in these regions following graduation (6). Experience of rural practice during an optometry student’s training has been deemed as one important element in increasing the number of optometrists taking up rural positions on graduation (6, 15). It is not known whether the differences between metropolitan and rural practice modulate the supervisory experience.

While there has been research focusing on medical and nursing student education and training, there is a dearth of studies investigating clinical placements in the allied health professions, particularly in the field of optometry. We previously described the experience of extended clinical placements in optometry from the students’ perspective (6). In order to understand what is working well and where areas of improvement are necessary, it is important to also gather insights from the optometrists who supervised the students. The current study aimed to describe the experience of extended clinical placements from the perspective of the optometrists who supervised students delivering care. Benefits and challenges associated with providing student placements for supervisors were investigated, as well as barriers and enablers to optometrist’s participating in the supervision of students. In particular, we examined whether rural optometrists viewed the extended placement more positively, and were more willing to take on a student for placement in the future.

## Methods

This study adhered to the tenets of the Declaration of Helsinki. The study design, recruitment, consent and procedures were approved by the Deakin University Human Research and Ethics Committee (HEAG-H 77 2018).

### Study design

An explanatory-sequential mixed methods study design was employed in this study (quantitative followed by qualitative). A mixed methodology was employed to add diversity and dimensionality to the findings. It was felt this would allow for deeper insights to be gained rather than using one method alone, adding breadth and richness to the data as well as rigour (16). The qualitative component of the study, which involved semi-structured telephone interviews, was undertaken after an online survey to ensure that factors influencing responses in the survey could be further explored. The survey items covered basic demographics and topics relating to: The benefits and challenges associated with extended placements; areas for improvement; the duration of the placement; and willingness to supervise students in the future (Appendix 1). The semi-structured interviews provided in-depth insights into supervising optometrist’s experiences and encouraged participants to expand upon and add to these *a priori* topics (Appendix 2).

### Setting, participants and recruitment

Since 2014, Deakin University’s Bachelor of Vision Science/Master of Optometry program has included a compulsory extended clinical placement in the final two terms of the Master of Optometry degree. It was the first optometry program in Australia to embrace community-based placements of an extended duration that are supplemented by several short-term placements at local health services, school-based clinics and University clinics. The extended placement is 26 weeks (2 x 13weeks), of which at least half must be in a non-metropolitan setting, or area where a shortage of optometrists has been reported (17). Students undertake placement four days a week in an accredited community practice anywhere in Australia or New Zealand.

Voluntary response sampling was utilised whereby all optometrists who had previously supervised a Deakin Optometry student on extended clinical placement during the period November 2014 to November 2018 (*n* = 499) were invited to participate. Recruitment was via email from a database at Deakin University of practitioners who had previously supervised students. Thirty-two emails were returned due to non-viable email address leading to a potential sample of 467 optometrists. The email provided a link to an online survey hosted in Qualtrics, (Provo, Utah, USA). Three reminder emails were sent. The email also provided a link to express interest in participating in a 20-minute telephone interview. This link was also included at the end of the survey. All optometrists who volunteered to participate were included in the study.

### Survey

The survey response period was from 7th August to 14th October 2018.

Review of the medical and allied health literature did not reveal an appropriate survey. Hence, one was constructed drawing on evidence from the medical literature as well as questions from Bentley et al’s (3) *Practitioner Perspectives on Extended Clinical Placement Programs for Students survey*. The survey was circulated amongst the research group for feedback and pilot tested on a sample of nine optometrists. The online survey comprised of four main sections and consisted of a total of 38 questions (Appendix 1).

1. Practitioner demographics
2. Supervision experience
3. Benefits and challenges associated with providing student placements
4. Willingness to supervise students in the future

Most survey questions were closed, however space was provided for additional comments in section 3 and 4.

Quantitative data were analysed using SPSS Version 26 (IBM, Armonk, New York, USA). Descriptive statistics were computed for demographics and each survey item. Differences in responses from metropolitan and rural practitioner groups were investigated using the chi-square test and Mann Whitney U Test. For the purpose of analysing differences between groups, participants who indicated they supervised a Deakin student in a major capital city or outer metropolitan area were pooled and the resultant group termed metro. Likewise, those who were based in large regional, small regional, rural or remote areas were pooled and the group termed rural. No participant who supervised students in a major capital city or outer metropolitan location also supervised students in any of the regional, rural or remote locations.

Associations between demographic variables and support for the extended clinical placement were analysed using the chi-square test. Where assumptions underlying the chi-square test were violated, likelihood ratios were performed (18). To analyse factors associated with willingness to supervise optometry students on extended placement again and the likelihood participants would recommend being a supervisor to a colleague, participants who indicated support by answering ‘yes’ or ‘maybe’ were combined and compared to those who answered ‘no’.

Analyses were two-tailed and *p*-values less than or equal to 0.05 were considered statistically significant.

### Interviews

The first author, an optometrist trained in qualitative techniques and interviewing, conducted the 20-minute telephone interviews between November 2018 and January 2019. The interviews followed a semi-structured interview guide of core open-ended questions with additional corresponding prompt questions to elucidate further detail (Appendix 2). Lines of questioning focused on the supervisor’s perspective of:

1. The benefits and challenges associated with the placement.
2. Areas for improvement.
3. The practitioners’ perspective of the duration of the placement.
4. Willingness to supervise students on extended placement in the future.

A qualitative descriptive approach was taken to respond to the aim of this study (16, 19). Rather than the development of theory or interpretive meaning, a rich, straight description of the participants experience was sought to be provided (20).

Each interview was audio-recoded before being transcribed verbatim by an external agency (Pacific Transcription, QLD Australia). For the purpose of data immersion and also accuracy, each transcript was audio-checked by the first author and a research assistant. Data were analysed in Nvivo Pro software, Version 11 (QSR International Pty Ltd, Doncaster, VIC Australia). Coding was completed independently by two researchers, one optometrist and one non-optometrist, and followed the 6-step method, the principles of inductive thematic analysis according to Braun and Clarke (21). To facilitate reflection and refinement, following initial analysis, the two researchers met to discuss similarities and differences in their analysis. Mind-mapping was then used to further refine themes. A final report of themes was generated following discussion with the rest of the research team. To illustrate each theme, quotations are used and cite the participant number. Ellipses were used to shorten long quotations.

To ensure methodological rigor, a number of techniques were utilised (16, 22-24). During qualitative data collection, rapport, trust and empathy established credibility (23). To ensure an understanding of terminology and culture, interviews were conducted by an optometrist. This made it easier for appropriate follow up questions to be asked. Participants were encouraged to share all views regardless of whether they were deemed positive or negative, with confidentiality, respect and trust re-enforced (23). To ensure credibility, dependability and confirmability, the first author maintained an audit trail and kept detailed notes (23, 24). Reflexive notes that detailed the first author’s thoughts, questions and prompts were used to record data collection and analysis decisions. The author also used these notes to consider how their background and underlying presumptions may potentially influence analysis.

Analysis of interview data was conducted by two researchers to allow for greater diversity as each had differing backgrounds and disciplinary expertise (22); the first author, an optometrist who had a thorough understanding of the specific placement; and the research assistant, who had no experience of student placements in a healthcare setting and a background in international relations and research. The two researchers regularly met to discuss the data and how their own background and experiences influenced their interpretation, ultimately adding to their understanding and producing joint interpretation (25). Finally, to provide transferability, the context, location, people studied are described in detail (24).

## Results

### Survey results

#### Survey participant characteristics

Responses were received from 46 optometrists who had supervised at least one Deakin optometry student on extended clinical placement representing a response rate of 10 per cent. 43 optometrists completed the survey in full. Participant characteristics are provided in Table 1.

**Table 1:** Participant characteristics

Characteristic	Number of responses			Overall percentage
Age	Male (%)	Female (%)	Other (%)	
20 to 29	1 (3)	3 (20)	1 (50)	11
30 to 39	12 (41)	3 (20)	0 (0)	33
40 to 49	7 (24)	5 (33)	1 (50)	28
50 to 59	4 (14)	4 (27)	0 (0)	17
60 to 69	5 (17)	0 (0)	0 (0)	11
Total	29 (100)	15 (100)	2 (100)	100
Years of practice	Male (%)	Female (%)	Other (%)	Overall percentage
1 to 5 years	0 (0)	2 (13)	0 (0)	4
6 to 10 years	4 (14)	2 (13)	0 (0)	13
11 to 15 years	6 (21)	0 (0)	1 (50)	15
16 to 20 years	4 (14)	6 (40)	1 (50)	24
21 to 25 years	6 (21)	3 (20)	0 (0)	20
26+ years	9 (31)	2 (13)	0 (0)	24
Total	29 (100)	15 (100)	2 (100)	100

**Fig. 1:**
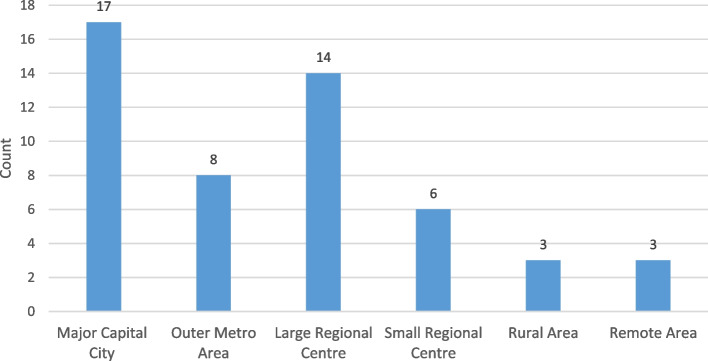
Geographic location of supervision of Deakin university students on extended clinical placement. Note that participants could select more than one response

Participants were generally key decision makers in the practice (59 per cent, 27 of 46) rather than employees or locum optometrists. Supervision of care delivery most often occurred in a major capital city or large regional centre as evident in Figure 1. Supervision generally occurred in a corporate setting (43 per cent, 20 of 46) followed by an independent (33 per cent, 15 of 46), franchise (20 per cent, 9 of 46) and other (four per cent, 2 of 46) setting.

#### Experience of supervising a student on extended clinical placement

As shown in Table 2, when asked to select the three main reasons the participant believed the practice agreed to supervise a Deakin optometry student on extended placement, the majority of participants reported that they believed the practice hosted a student to provide them with a positive learning experience, to recruit new graduates or give back to the profession. Only 1 out of 46 participants identified financial motivations as being a primary driver for taking on a student.

**Table 2:** The three main reasons participants believed the practice agreed to host a Deakin student on extended clinical placement. Note that participants could select up to three responses

**Reasons for hosting a student**	**Metro (n)**	**Rural (n)**	**Total (n)**
Had an existing connection to the student	6	3	9
To recruit graduates	13	12	25
To provide students with a positive learning experience	22	14	36
To gain extra help around the practice	1	3	3
To gain extra help with patients	3	2	5
To increase revenue	1	0	1
To give back to the profession	15	9	24
To be affiliated with the university	5	2	7
To develop or enhance teaching skills	5	4	9
To gain knowledge on the latest developments	2	2	4
To provide variety in clinical work	5	4	9

**Fig. 2:**
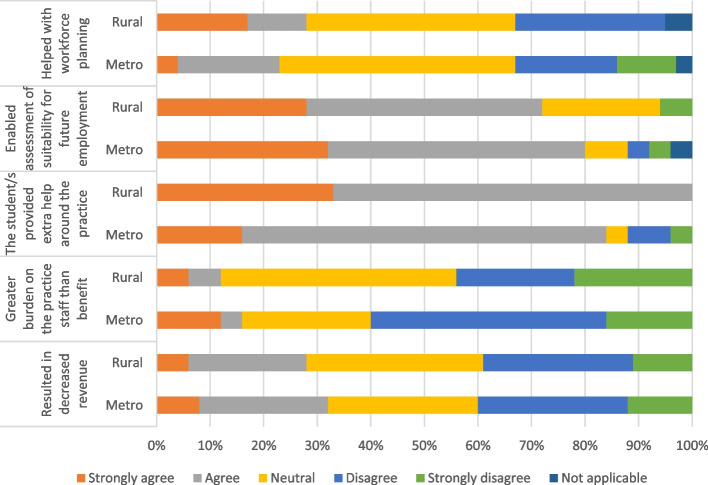
The Impact of supervising the student on the practice setting

As shown in Figure 2, when asked whether supervising the student resulted in a change in revenue, 30 per cent (13 of 43) of participants felt there was a negative financial impact of hosting a student. In contrast, 87 per cent of participants (40 of 45) believed the student provided extra help around the practice (Figure 2). Furthermore, 77 per cent of participants (33 of 43) felt that hosting the student was beneficial as it enabled the assessment of suitability for future employment (Figure 2).

**Fig. 3:**
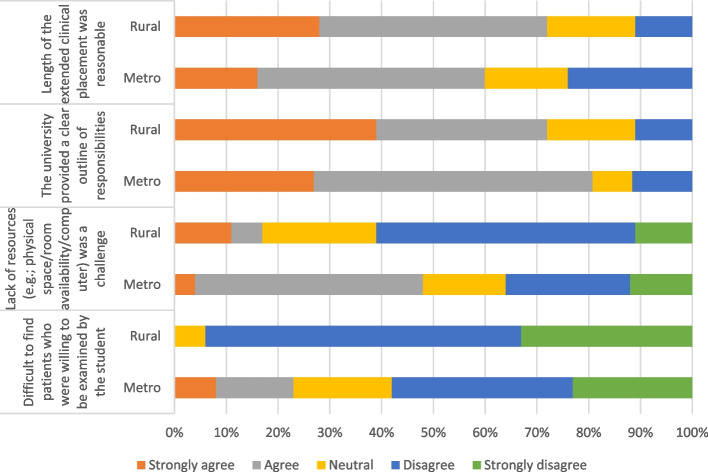
The impact of supervising the student on operational factors

The majority of participants responded that it was not difficult to find patients willing to be examined by the student (73 per cent, 32 of 44, as shown in Figure 3). However, lack of resources in terms of physical space, room availability or a computer was found to be a challenge for 35 per cent of participants (15 of 43). As evident in Figure 3, 65 per cent of participants (28 of 43) felt that the length of the placement was reasonable.

**Fig. 4:**
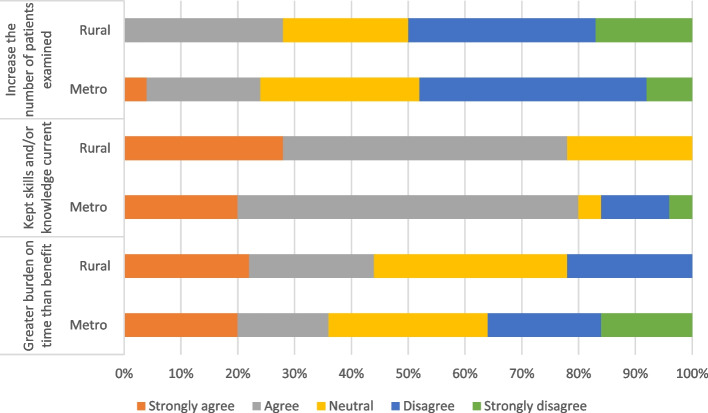
The impact of supervising the student on the supervising optometrist

The majority (79 per cent, 34 of 43) of participants believed supervising the student kept their skills and knowledge current (Figure 4). For 40 per cent of participants (17 of 43) this came with a greater burden on time than benefit (Figure 4).

**Fig. 5:**
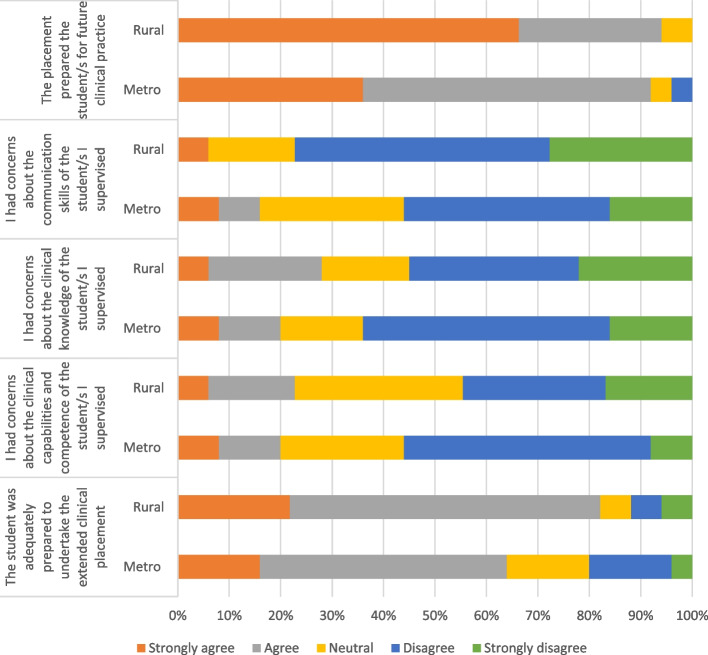
Student capabilities and preparedness

Most (72 per cent, 31 of 43) participants felt that the student was adequately prepared to undertake the extended clinical placement as shown in Figure 5. Only a small number of participants had concerns about the clinical capabilities, competence, clinical knowledge and communication skills of the students (Figure 5). Overall, the vast majority (93 per cent, 40 of 43) of participants believed that the placement prepared student for future clinical practice.

**Fig. 6:**
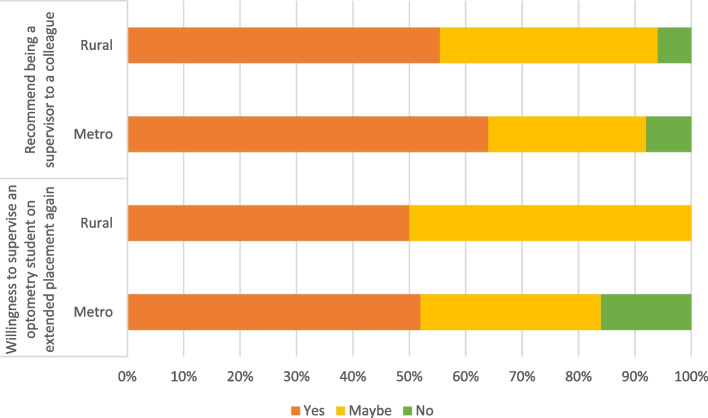
Support for extended placements

The majority of participants were open to supervising further students with 53 per cent (23 of 44) responding yes when asked and 40 per cent (17 of 44) responding maybe as indicated in Figure 6. Furthermore, when asked whether they would recommend being a clinical supervisor of students to a colleague, 60 per cent (26 of 44) responded yes while 30 per cent (13 of 44) responded maybe (Figure 6).

#### Comparison of metropolitan-based supervisors versus rural-based supervisors

Practitioners in metropolitan locations found it more difficult to find patients willing to be examined by students than their rural based colleagues (U = 299.0, *p* = 0.050). In all other aspects of the experience of hosting a student, no statistically significant differences were found between metropolitan and rural groups. Likewise, no significant differences were found with regards to the reasons for hosting students.

#### Factors associated with willingness to supervise further students and likelihood participants would recommend being a supervisor to a colleague

There was a significant association between the number of students participants had previously supervised and willingness to supervise students in the future. Participants who had supervised more than seven students, from any university, were less willing to supervise further students than participants who had supervised between one to six students (likelihood ratio (3, *N* = 44) = 6.119, *p* = 0.013). There were no significant associations between willingness to supervise students in the future and gender, age, years of practice, geographic location, mode of practice, or the optometrists’ role in the practice .

Males were significantly more likely to recommend being a clinical supervisor of students to a colleague (likelihood ratio (2, *N* = 44) = 11.045, *p* = 0.004), as were, those who had previously supervised a Deakin student in an independent setting (likelihood ratio (1, *N*=44) = 4.494, *p* = 0.034). However, participants who were an employee or locum optometrist were significantly less likely to recommend being a supervisor of students to a colleague (likelihood ratio (1, *N* = 44) = 8.104, *p* = 0.004). There were no statistically significant associations between likelihood of recommending to a colleague supervising a Deakin student and age, years of practice, or geographic location.

### Interview results

A total of nine interviews were conducted, with different participants to those that participated in the pilot study of the survey. Two of the nine participants interviewed were female. Two participants were in the age category 30 to 39 years, four were in the 40 to 49 years, two in the 50 to 59 years and 1 in the 60 to 69 years. Three participants were from a major capital city, two each from an outer metropolitan area, large regional centre and small regional centre. The length of each interview varied, with the longest duration being 42 minutes and the minimum being 21 minutes.

The four predominant themes which emerged are summarised in Table 3.

**Table 3:** Themes and subthemes: Supervisor perspectives

**Theme**	**Subthemes**
Supervision encourages reflection	• Improves confirmation bias • Sense of greater accountability
Continuity is key	• Where immersion is fostered, stronger relationships are built • Employment trial
The sizable commitment of placement	• Demanding, fatiguing experience • Student preparedness • Lack of financial reimbursement and sustainability of placements
Mentoring through leadership	• The feel-good factor in supervision • Maintaining the future of the profession • Learning is an ongoing process

#### Theme 1: supervision encourage reflection

Repeatedly, supervisors articulated that the experience of supervising the student prompted them to reflect on their own practice. They described how the process of internally examining and critically assessing their methods of practice was only triggered by the presence of the student, who often asked questions they had not previously considered. In addition, supervisors expressed feeling a sense of accountability and renewed desire to practice in an evidence-based manner.

*P9 “You have to make sure you’re practicing what you preach. And I think that’s actually been very useful for me because it’s allowed me to reflect on some of the things that—well perhaps I was doing shortcut.”*

The potential for deeper understandings and new knowledge to be gained from the student was very much appreciated by the supervisors.

*P2 “I’ve actually learned stuff from the students…That process of teaching someone and explaining crystallises thoughts in your head that you’d not joined up.”*

The process assisted them to identify their own knowledge deficits and helped them realise their tendency to unknowingly be susceptible to cognitive biases such as availability, confirmation or anchoring.

P6 “*Sometimes you can get sort of one tracked. I’d say, oh yeah, it’s definitely that but then you haven’t considered all the possibilities that you would as a student.”*

Ultimately, supervisors described how this heightened awareness on their practice improved their clinical performance, thereby improving patient care.

*P4 “I’m a better optometrist because I have students.”*

#### Theme 2: continuity is key

Supervisors described the extended duration of the placement as being the key element which fostered truly immersive, formative experiences. They consistently reiterated that the students required time to ‘settle in’ before they could focus on their learning.

*P9 “It’s good because it provides continuity. Yes, it’s nice to get variety, as in different practitioners’ views and different settings. I think that’s important. But I think the efficiency out of having a longer period is much more beneficial, because once you form that relationship with the student, then the teaching and learning can happen in a very natural way.”*

*P8 “I always say it takes about 3 months for them to get used to the environment. The change from academic to community happens at probably that two or three month mark, where they’re confident in their new environment now.”*

For the supervisors, the longer duration meant repeated interactions and ongoing observation of students’ skills occurred, resulting in them building trust in the students’ competence.

*P9 “If it’s a shorter placement, I think you tend to be much more interventionalist, as in- ‘what are you doing here and why?’ because you’ve not had that time to scope them out. You’re much more likely to say ’just watch me’ because it’s easier.”*

Supervisors felt that the students became more useful to them overtime as their confidence and skills matured. The vast majority of supervisors who had experience with shorter duration placements reported finding them more disruptive to their practice and businesses.

*P1 “From a practice point of view, six months is a long time but it’s actually less of an impact the longer they’re there. It’s the short impact stays that often are the ones that disrupt you the most….whereas the longer they’re there….they’re just part of your business as opposed to having to be an imposition on you.”*

Patient care was felt to be improved as learning and service provision could be integrated.

*P9 “If you see something and you want to deal with it on the day, you can use the student to continue some of the investigations. So it actually has improved efficiency in certain areas.”*

Interestingly, supervisors from rural areas felt that it was easier for them to provide the student with a diverse range of clinical scenarios to experience compared to their urban based colleagues.

P3 “*We’re lucky here in (small regional centre) as we get a lot of interesting cases sent to us from the (local hospital). They wouldn’t get this good experience if they only did placement in a city.”*

Supervisors highlighted that the extended placements more closely represented the conditions in which students were likely to find employment. They described the placement as a transitional phase for the students into the workforce.

*P1 “Being able to do it for six months as opposed to a small period actually lets them assimilate into real-world optometry outside of university.”*

A major incentive for participating in the placement from the supervisors’ point of view was the recruitment opportunity it offered. This was particularly pertinent for the supervisors in rural locations as they described how important it was for the student to experience life in the region and make a determination as to whether they felt that they could live in that location on a long-term basis.

*P1 “I want someone to be able to see themselves work and live in our community for an extended period of time. I really want them to actually want to be here in our little town.”*

*P3 “It’s the opportunity to size each other and the town up, over more than just a couple of weeks. To see if the student can see themselves fitting in.”*

The opportunity to mentor the student in their environment and train them in the manner they wished was seen to be of great benefit.

*P7 “The fact is you are nurturing them in your environment, so they take on your culture, your environment.”*

#### Theme 3: the sizeable commitment of supervision

Guiding the student towards independence was described as a time demanding process, with some supervisors expressing that the early stage was the most stressful period. However, where competence was observed and trust developed, this progressed to less time-demanding supervision and more active student participation.

*P4 “You spend your first two months - babysitting is too strong a word - but it’s taking a lot of your time. The second two months they can sort of be let free but that’s when they start to have an impact on your book a bit more because of the timing they require. Final two months, they’re beneficial and helpful and it’s like you’ve got another optometrist in the practice pretty much.”*

The majority of supervisors articulated that although the initial period of placement was a challenging time in clinical supervision, it was an expected and necessary transitional phase. It was clear however, that not all supervisors were comfortable with this demand, particularly in the context that some students were not believed to be as prepared as anticipated.

*P5 “Some of their skills were probably not as proficient as I thought,….just because their skills were slow and I thought they would be at a different level. Obviously as time went by they improve…but it made things difficult initially.”*

The supervisors detailed the challenge of wanting to provide the students with a full complement of clinical exposure but that this came with significant stress and time-cost.

*P5 “It was quite tiring and stressful, more so than I thought it would be because I was constantly being pushed for time. I didn’t want the student to not have the experience.”*

Likewise, some supervisors described the extent to which the students engaged in learning opportunities as varying according to the students’ skills, attitude, and personal aspirations.

*P4 “I had students who didn’t really respect the practice and I suppose, maybe didn’t even take the profession of optometry as seriously as they should.”*

While it was felt that the length of the placement overall was appropriate, one supervisor suggested the student placement working week would be better if it was reduced from four days a week to three days a week.

The lack of financial incentive or reimbursement was highlighted as a potential barrier for ongoing involvement in student supervision. One supervisor noted the inequity amongst professions in governmental financial support offered in Australia.

*P4 “I reckon it’s an interesting challenge for optometry as a profession and universities within the profession…..how do we make student placements sustainable for practitioners to put the time and effort into..….It’d be unreal if we saw some sort government subsidy model evolve where if most optometry students spend their last two to six months in practices that there’s some way that that gets recognised or made sustainable somehow for the practitioners.”*

#### Theme 4: mentoring through leadership

Overwhelmingly the supervisors described the experience of supervising the students as rewarding. Many felt that the duration of the placement was particularly pivotal in this as it enabled stronger relationships to be built and for student growth to be witnessed.

*P1 “I really did enjoy it. We supervise a lot of different students over different capacities, but I have probably enjoyed the Deakin students the most just because it was longer…. It’s great seeing students learn and grow up and gain new skills. It’s a positive experience for I think both practitioner and student.”*

All the supervisors spoke of having passion for their profession and seeing the importance in educating the next generation of optometrists.

*P7* “*You get that feel good factor- as you’re giving back to the profession and optometry”*

Many also described the beneficial impact it had on their businesses.

*P8 “The experiences as a supervising optometrist have been very fruitful for the business, without a shadow of a doubt. It’s bringing out the right cultures and the right professionalisms that our business needs.”*

Some described how over the course of their career, the manner in which optometry is practiced has changed. The ability to demonstrate by example to the student that learning is a life-long process was seen as beneficial.

P3 “*Hopefully they can learn that no matter how old you are, no matter how experienced you are, there’s always something new that you can actually learn, every day.”*

Likewise, many supervisors articulated that it was nice to have someone new in the practice, as it brought their enthusiasm for their profession back. This was particularly valued as they described how optometry can be an isolating career on a day-to-day basis.

*P7 “It’s good for me, it’s good for them. I love that vibrance, the youth in the practice…It freshens up the practice. It stops the practice from becoming stale because you’ve always got that new individual.”*

This was particularly the case for supervisors in rural areas where they otherwise had little ability to connect with their optometry colleagues. Likewise, rural supervisors described how it was difficult to attend continued education events in person, but that in hosting students they were exposed to contemporary ideas regarding optometry practice, which enabled them to have interactive educational experiences from near peers and then incorporate the latest knowledge and techniques into their existing practicing frameworks.

P4 “*I liked having someone who was almost my colleague, my peer, in the practice. It can be lonely out here in (small regional centre) as the only eye care practitioner. It was nice to share knowledge.”*

Supervisors overwhelmingly described patients as being receptive to the student’s presence. Many practitioners even described having the student present as having a ‘business building aspect’ as it demonstrated to the patients that the practice was to be held at higher esteem.

*P4 “We’ve had feedback from patients in the past saying they were really impressed by the way the individual optometrist was teaching and the relationship that the optometrist had with the student. That enhances that optometrists’ perception in the mind of their patients.”*

*P8 “They kind of appreciate it—it brings more value to our clinical professionalism image to be honest…They know that the pedigree of professionalism in this store is at a different level.”*

## Discussion

To the best of our knowledge, this is the first-time supervising optometrists’ experiences of clinical placements have been reported. Understanding the experience of supervising practitioners is necessary for the advancement of placement programs. Deakin University’s Optometry program was the first program in Australia to establish an extended clinical placement of 26-weeks. It is therefore appropriate an evaluation of the effectiveness and impact of such a placement is undertaken. Capturing the experience of the placement from the supervising practitioner’s perspective is one key element in this evaluative process.

This study has demonstrated that the experience is an overall positive one from the perspective of the supervising optometrists’ who have overseen the students care delivery. A previous investigation undertaken by Bentley et al. in 2016, which examined optometric professionals’ views on the concept of extended student clinical placements, found the perceived barriers to taking on a student for extended placement included lack of time, financial remuneration and space (3). With reference to the lack of financial remuneration, while the analysis in this study demonstrated that there are minimal financial barriers to taking on a student, the supervisors did highlight that they believed their efforts and contributions should be rewarded and acknowledged through financial incentives. Whether there is the potential for practitioners to receive remuneration for teaching, much like the Australian Federal Government sponsored Practice Incentives Program Teaching Payment available to General Practitioners, needs to be raised with governing bodies.

In our previous investigation of student perspectives of extended clinical placements, students highlighted that the familiarity offered by remaining in the same setting for an extended period provided a better learning environment where they could concentrate on their clinical training (6). In that study, the students deemed the length of the 26-week placement to be appropriate. Participants in the Bentley et al., 2016 study preferred a relatively short extended clinical placement program, with those identified as key decision makers in the business seeking to host a student for no longer than 10 weeks. However, the current study found that most participants felt that the duration, of 26-weeks (2 x 13 weeks), was acceptable, with 81 per cent of participants either agreeing or neutral when asked whether the placement length was reasonable. From the supervisor’s perspective it appears that the 2 x 13-week placements allowed the student time to be accepted as a team member and begin to become an asset to the practice rather than a burden, which is recognised as being a factor that facilitates effective learning (26). Similar to the medical literature, the supervisors distinguished between a turning point where the student became ‘fruitful’ for the business - a time point when the students generate sufficient benefit to counter the initial time and effort burden (27). Further research is needed before a consensus on the optimal duration of placement can be reached.

Overwhelmingly, practitioners cited altruistic reasons for taking on a student, reporting they had a strong desire to ‘give back’ to their profession and sought to provide students with a positive learning experience. Similar personal and professional motivating factors were found in a study focusing on understanding why rural general practitioners supervise registrars (28). This knowledge may prove helpful for informing the recruitment of future supervising practitioners. In the present study, the practitioners felt rewarded by the mentorship opportunity, and relished in the chance to mould the next generation of optometrists. Nevertheless, they did describe tensions between the demands of clinical practice and the commitment to teaching, particularly early in the placement when the student’s skills may not have been up to the standard that they expected. The supervisors described developing a situation in the latter part of the placement where patient care and student teaching were no longer seen as competing activities but rather, each contributing to the performance of the other. This finding is consistent with other studies which have found teaching in an extended placement model leads to greater job satisfaction compared to teaching in short block placements, and less tensions between teaching and delivery of patient care (7, 9, 29). Interestingly, the findings demonstrated that the more students a participant had previously supervised, the less likely they were to be willing to supervise further students. This likely suggests an element of ‘burn-out’ associated with clinical supervision.

Our student perspectives investigation revealed a complex dynamic between student and supervisor, and the importance of having a positive relationship from the student’s point of view (6). In the case of supervisors and students, power imbalances were present and referred to the supervisors having authority and control over the students. Our previous study highlighted that elements of conflict-management and power imbalance were a concern for a number of students. Such instances were not raised by supervisors in the present study. This is not surprising as those who hold the balance of power may be dismissive of the effect of this imbalance on others or, may be unaware of the power imbalance in the first instance. In the present study, supervisors predominantly described having a collegial relationship with their student, perhaps further evidence that supervisors are unaware of the power differentials between student and supervisor. Further research which explores the psychological and professional dynamics involved in the student-supervisor relationship is required.

Extended placements are part of the strategy to increase the rural and remote healthcare workforce by providing a ‘context-specific, community engaged education’ (8). Survey data in the present study showed no evidence rural supervisors were more likely to host students, or that the experience was particularly different for rural supervisors in comparison to their metropolitan based colleagues. This was in contrast to interview data where the rural supervisors reported benefits to hosting a student that metropolitan-based optometrists did not. For instance, during the interviews, rural supervisors described a willingness to use the placement as an opportunity to recruit the students to their rural locations. As the student-supervisor relationship developed, the supervisors came to value the contributions of their students. For the supervisors based in rural areas this potential for near-peer knowledge sharing was highlighted as particularly beneficial as professional isolation was cited as common in such areas. While this near-peer learning may not have been unique to the rurally located optometrists, metropolitan-based optometrists have easier access to interactive continuing professional education. During the interviews, the rural based supervisors consistently mentioned believing that they could offer students a superior learning experience as they could provide a greater diversity in clinical experiences and exposure to more advanced disease. In addition, the survey data demonstrated that the rural based supervisors were less likely to report difficulty finding patients willing to be examined by the student compared to the metropolitan based supervisors. Perhaps this speaks to the welcoming nature often used to describe rural communities. This reiterates the views of students who felt that placements in rural locations gave them exposure to a more diverse range of pathologies and that rural communities were more friendly and welcoming (6). Future research is needed to determine whether there are actual differences during clinical placement between rural and metropolitan based clinical settings in terms of the comparative cases managed, and whether indeed a rural clinical placement does offer a more effective and worthwhile learning experience.

Concerns have been raised about the increasing reliance on community-based education in medical programs, and the subsequent dependence on the altruism of the practitioners who supervise students (30, 31). There is limited evidence in the medical literature that considers what specifically is needed for a LIC to be sustainable over time (8). The long-term sustainability of such placements has not yet been investigated in the optometry field. With increasing student numbers and demand, whether community practitioners will remain willing to provide placement experiences is yet to be seen. How to recruit and retain skilled optometry supervisors is worth consideration particularly as there is currently no financial remuneration available for supervising students. How many students can realistically be accommodated in these forms of placements is a complex question and depends not only on the number of supervisors but also their quality, the size of the practice and the patient population (31, 32).

## Limitations

It may be difficult to generalise the findings of this study as it was conducted using a single institution clinical placement. It is also unlikely that strong negative views would have been captured as those with such an experience are not likely to engage with a study conducted by the same institution. Similarly, those who participated are likely to be more invested in the training of students than those who did not respond to their invitation to participate. This is particularly the case with the interviews.

## Conclusion

The experience of hosting an optometry student on extended placement is overall a positive one. However, extended placements impose a time cost on supervisors and in contrast to medical training programs, attract no financial incentives. There are minimal differences in the benefits or challenges associated with hosting a student for supervisors in rural locations compared with those in metropolitan locations and overall, the level of support for extended placements was the same regardless of the supervisor's practice location. Rural supervisors believe they offer students a superior learning experience when compared to their metropolitan based colleagues. Rural supervisors also believed the placement gave them more of an opportunity to recruit graduates. University schools of optometry might carefully consider engaging in discussion about the duration of such placements, but 26 weeks was considered appropriate by supervisors. These findings will inform the evolution of effective clinical training programs in optometry and provide a case for the establishment of such models within other health professions.

## Supplementary Information

Below is the link to the electronic supplementary material.

**Additional file 1.**

**Additional file 2.**

## Data Availability

The datasets generated and analysed during the current study are not publicly available due to the sensitive nature of the data and the consent provided for participation but are available from the corresponding author on reasonable request.

## Abbreviations

LIC: Longitudinal Integrated Clerkships
