# Supplementary material for: Supervisor experiences of extended clinical placements in optometry: a mixed methods study
Source: BMC Med Educ. 2022 Dec 9;22:854. doi: 10.1186/s12909-022-03918-2 (PMC9733108; doi:10.1186/s12909-022-03918-2)
Supplement: Supplementary file 1 — Additional file 1. [file 12909_2022_3918_MOESM1_ESM.docx]

APPENDICES 1

**Supervisor experiences of extended clinical placements in optometry: A mixed methods study**

Authors

Jacqueline M Kirkman, MOptom, BVisSci^1^

Sharon A Bentley, PhD, MOptom, MPH, BScOptom FACO, FAAO^2^

Ryan J Wood-Bradley, PhD, BSc (Hons)^1^

Craig A Woods, PhD, BSc (Hons) FACO, FAAO^3^

James A Armitage, PhD, MOptom, BSc (Optom) FACO, FAAO^1^

^1^Deakin Optometry, School of Medicine, Deakin University, Waurn Ponds, Australia

^2^School of Optometry and Vision Science, Queensland University of Technology, Kelvin Grove, Australia

^3^School of Optometry and Vision Sciences, University of New South Wales, Sydney, Australia

Corresponding author: Jacqueline Kirkman [jac.kirkman@deakin.edu.au](mailto:jac.kirkman@deakin.edu.au)

**Survey Questions**

**Category 1: Practitioner demographics**

1. What gender are you?
   1. Male
   2. Female
   3. Other
2. What is your age group?
   1. 20 to 29
   2. 30 to 39
   3. 40 to 49
   4. 50 to 59
   5. 60 to 69
   6. 70 +
3. How many years have you been practicing optometry?
   1. 1 to 5 years
   2. 6 to 10 years
   3. 11 to 15 years
   4. 16 to 20 years
   5. 21 to 25 years
   6. 30 + years
4. In what geographic location(s) do you currently practice? Check all those that apply
   1. A large Capital City (population >350,000 I.e.; all Australian cities, except Darwin and Hobart)
   2. Outer Metropolitan area or Small Capital City (population 100,000 to 349,999 E.g.; Geelong, Hobart, Darwin)
   3. Large Regional Centre (population 25,000 to 99,000)
   4. Small Regional Centre (population 10,000 to 24,999)
   5. Rural Area (population 5,000 to 10,000)
   6. Remote Area (population <5000)

**Category 2: Practitioners’ previous involvement in supervision of optometry students**

1. How many optometry students **in total** have you previously supervised delivering care?
   1. 1 to 3 students
   2. 4 to 6 students
   3. 7 to 9 students
   4. 10 + students
2. Which university have the optometry students you have previously supervised been from? Check all those that apply.
3. UNSW
4. The University of Melbourne
5. QUT
6. Flinders University
7. Deakin University
8. University of Auckland
9. Other overseas University
10. In which mode of practice have you supervised optometry students delivering care? Check all those that apply.
11. Independent
12. Corporate
13. Franchise
14. Public eye-care institution
15. Optometry teaching institution
16. Other (please specify)
17. Which of the following best describes your role in the practice where you have supervised students delivering care? Check all those that apply.
18. Employee or locum optometrist
19. Owner or partner optometrist
20. Managing or administrating optometrist
21. In what geographic location(s) have you supervised students delivering care? Check all those that apply.
22. A large Capital City (population >350,000 I.e.; all Australian cities, except Darwin and Hobart)
23. Outer Metropolitan area or Small Capital City (population 100,000 to 349,999 E.g.; Geelong, Hobart, Darwin)
24. Large Regional Centre (population 25,000 to 99,000)
25. Small Regional Centre (population 10,000 to 24,999)
26. Rural Area (population 5,000 to 10,000)
27. Remote Area (population <5000)
28. In total, how many Deakin optometry students have you supervised on an extended clinical placement? For the purposes of this survey an extended clinical placement is defined as a placement a student undertakes for a continuous period, ranging between 11 to 26 weeks, for at least 4 days per week.
29. 1 to 3
30. 4 to 6
31. 7 to 9
32. 10 + students
33. In what year/s did you supervise a Deakin optometry student on extended clinical placement? Check all those that apply.
34. 2014
35. 2015
36. 2016
37. 2017
38. 2018
39. 2019
40. 2020
41. How many Deakin optometry students on extended clinical placement you have supervised at the **same** time?
42. 1 student at a time
43. 2 students at a time
44. 3 students at a time
45. Other, please specify
46. In which mode of practice have you supervised Deakin optometry student on extended clinical placement? Check all those that apply.
47. Independent
48. Corporate
49. Franchise
50. Other (please specify)
51. In what geographic location(s) have you supervised Deakin optometry student on extended clinical placement? Check all those that apply.
52. A large Capital City (population >350,000 E.g.; all Australian cities, except Darwin and Hobart)
53. Outer Metropolitan area or Small Capital City (population 100,000 to 349,999 E.g.; Geelong, Hobart, Darwin)
54. Large Regional Centre (population 25,000 to 99,000)
55. Small Regional Centre (population 10,000 to 24,999)
56. Rural Area (population 5,000 to 10,000)
57. Remote Area (population <5000)
58. What are the main reasons you believe the practice agreed to supervise a Deakin optometry student on extended clinical placement? Please select the top 3 reasons, with 1 being the reason most strongly associated, and so on.
59. Had an existing connection to the student
60. To recruit graduates
61. To provide students with a positive learning experience
62. To gain extra help around the practice
63. To gain extra help with patients
64. To increase revenue
65. To give back to the profession
66. To be affiliated with the university
67. To develop or enhance teaching skills
68. To gain knowledge on the latest developments
69. To provide variety in clinical work
70. Are there any other reasons you agreed to supervise a student?

Comments:

**Category 3: Benefits and challenges associated with providing student placements**

Reflecting on your time supervising a Deakin optometry student/s on extended clinical placement, please answer the below questions. Please feel free to provide any additional comments following each statement.

1. Supervising students was beneficial as it enabled assessment of suitability for future employment.
   1. Strongly agree
   2. Agree
   3. Neutral
   4. Disagree
   5. Strongly disagree
   6. Not applicable

Comments:

1. It was difficult to find patients who were willing to be examined by the student/s.
   1. Strongly agree
   2. Agree
   3. Neutral
   4. Disagree
   5. Strongly disagree

Comments:

1. Supervising the student/s kept my skills and/or knowledge current.
   1. Strongly agree
   2. Agree
   3. Neutral
   4. Disagree
   5. Strongly disagree

Comments:

1. Supervising the student/s had a greater burden on time than benefit.
   1. Strongly agree
   2. Agree
   3. Neutral
   4. Disagree
   5. Strongly disagree

Comments:

1. Supervising the student/s was beneficial as it helped with workforce planning.
   1. Strongly agree
   2. Agree
   3. Neutral
   4. Disagree
   5. Strongly disagree
   6. Not applicable

Comments:

1. Supervising the student/s had a greater burden on the practice staff than benefit.
   1. Strongly agree
   2. Agree
   3. Neutral
   4. Disagree
   5. Strongly disagree

Comments:

1. Supervising the student/s resulted in decreased revenue.
   1. Strongly agree
   2. Agree
   3. Neutral
   4. Disagree
   5. Strongly disagree

Comments:

1. The student/s provided extra help around the practice.
2. Strongly agree
3. Agree
4. Neutral
5. Disagree
6. Strongly disagree

Comments:

1. Supervising the student/s enabled me to increase the number of patients examined.
   1. Strongly agree
   2. Agree
   3. Neutral
   4. Disagree
   5. Strongly disagree

Comments:

1. Lack of resources (e.g.; physical space/room availability/computer) was a challenge when supervising the student/s.
   1. Strongly agree
   2. Agree
   3. Neutral
   4. Disagree
   5. Strongly disagree

Comments:

1. I had concerns about the clinical capabilities and competence of the student/s I supervised.
   1. Strongly agree
   2. Agree
   3. Neutral
   4. Disagree
   5. Strongly disagree

Comments:

1. The student was adequately prepared to undertake the extended clinical placement.
2. Strongly agree
3. Agree
4. Neutral
5. Disagree
6. Strongly disagree

Comments:

1. The university provided a clear outline of my responsibilities as a supervisor.
2. Strongly agree
3. Agree
4. Neutral
5. Disagree
6. Strongly disagree

Comments:

1. I had concerns about the clinical knowledge of the student/s I supervised.
2. Strongly agree
3. Agree
4. Neutral
5. Disagree
6. Strongly disagree

Comments:

1. I had concerns about the communication skills of the student/s I supervised.
2. Strongly agree
3. Agree
4. Neutral
5. Disagree
6. Strongly disagree

Comments:

1. The university’s support to staff supervising students on placement was appropriate.
2. Strongly agree
3. Agree
4. Neutral
5. Disagree
6. Strongly disagree

Comments:

1. The length of the extended clinical placement was reasonable.
2. Strongly agree
3. Agree
4. Neutral
5. Disagree
6. Strongly disagree

Comments:

1. The placement prepared the student/s for future clinical practice.
   1. Strongly agree
   2. Agree
   3. Neutral
   4. Disagree
   5. Strongly disagree

Comments:

**Category 4: Willingness to supervise further students**

1. Would you be willing to supervise a Deakin optometry student on extended placement again?
2. Yes
3. No
4. Maybe

Please comment on the reasons for your response:

1. Would you recommend being a clinical supervisor to a colleague?
2. Yes
3. No
4. Maybe

Please comment on the reasons for your response:

1. What was the best part of the placement from your perspective?

Please provide comments:

1. How could the placement be improved?

Please provide comments:
