# Supplementary material for: Supervisor experiences of extended clinical placements in optometry: a mixed methods study
Source: BMC Med Educ. 2022 Dec 9;22:854. doi: 10.1186/s12909-022-03918-2 (PMC9733108; doi:10.1186/s12909-022-03918-2)
Supplement: Supplementary file 2 — Additional file 2. [file 12909_2022_3918_MOESM2_ESM.docx]

APPENDICES 2

**Supervisor experiences of extended clinical placements in optometry: A mixed methods study**

Authors

Jacqueline M Kirkman, MOptom, BVisSci^1^

Sharon A Bentley, PhD, MOptom, MPH, BScOptom FACO, FAAO^2^

Ryan J Wood-Bradley, PhD, BSc (Hons)^1^

Craig A Woods, PhD, BSc (Hons) FACO, FAAO^3^

James A Armitage, PhD, MOptom, BSc (Optom) FACO, FAAO^1^

^1^Deakin Optometry, School of Medicine, Deakin University, Waurn Ponds, Australia

^2^School of Optometry and Vision Science, Queensland University of Technology, Kelvin Grove, Australia

^3^School of Optometry and Vision Sciences, University of New South Wales, Sydney, Australia

Corresponding author: Jacqueline Kirkman [jac.kirkman@deakin.edu.au](mailto:jac.kirkman@deakin.edu.au)

**Semi-structured interview guide**

How would you describe your experience supervising a student on extended placement?

- What impact did supervising the student/s have on your ability to deliver care?
- What impact did supervising the student/s have on your patients?
- What impact did supervising the student/s have on your practice?
- What impact did supervising the student/s have on you?

What benefits do you see to supervising a student?

- What factors do you believe would enable/help during these placements?

What challenges did you face when supervising a student?

- What factors do you see act as barriers during these placement?

How do you feel the extended placement could be improved?

Do you think it is a good experience for the students?

- What impact has the placement experience had on students?
- Overall, what has worked well?
- Overall, what hasn’t worked well?

What would strengthen or improve the placement experience for the students?

- What barriers do you believe there are for the students attending the placement?
- What factors do you see could better enable students during the placement?

How do you feel about the duration of the placement? Please Elaborate.

- Did the student/s remain with you for 3 or 6 months? What pros/cons do you see to this?
- Do you feel that the duration gave the student a chance to be immersed in the practice?
- Ideally how long to you believe the placements should run? What are the reasons for this?

Would you supervise a student again on an extended placement?

- Could you elaborate for me the reasons why or why not?
- Would you recommend being a clinical supervisor to a colleague? Elaborate.
